# The use of adjuvant anteromedial support plate fixation in the treatment of comminuted proximal humerus fractures: A retrospective observational study

**DOI:** 10.1097/MD.0000000000047144

**Published:** 2026-01-16

**Authors:** Zhengfeng Mei, Wentao Lei, Guobiao Pan, Lingzhi Ni

**Affiliations:** a Department of Orthopaedics, Hangzhou Third Hospital Affiliated to Zhejiang Chinese Medical University, Hangzhou, Zhejiang, China.

**Keywords:** internal fixation, proximal humeral fractures, shoulder joint, support plate

## Abstract

Proximal humerus fractures (PHFs) are clinically common and are usually treated with proximal lateral locking compression plate fixation. However, for partial comminuted PHFs, lateral plate fixation cannot restore the support of the proximal humerus medial column, so there are more postoperative complications and even surgical failure. To improve the success rate of proximal humeral comminuted fracture surgery and reduce the complications, we used anteromedial support plate combined with lateral locking compression plate for the treatment of proximal humeral comminuted fracture. From January 2017 to April 2023, 31 cases of comminuted PHFs were treated, of which 17 cases were treated with lateral locking compression plate of proximal humerus, and 14 cases were treated with anteromedial support plate combined with lateral locking compression plate of proximal humerus. The operative time, intraoperative bleeding loss, postoperative visual analogue scale score, postoperative Constant–Murley score, fracture healing time, operative complications, and internal fixation were compared between the 2 groups. There were no statistical difference in preoperative general data between the 2 groups. All cases were followed up from 6 to 24 months, with an average of 14.50 months. There was significant difference in fracture healing time between the 2 groups. No significant differences: operation time, intraoperative beding loss, visual analogue scale score 3 months after surgery, and Constant–Murley score 8 months after surgery. Complications: 2 cases in the double plate group; 5 cases in the single plate group. The excellent and good rate was 78.57% (11/14) in double plate group and 76.47% (13/17) in single plate group. In the comminuted PHF, the anteromedial support plate can reconstruct the support function of the internal humerus column, improve fracture stability, reduce complications, and facilitate the functional recovery of the shoulder joint. The postoperative effect is satisfactory. However, the number of clinical cases in the group is small, the follow-up time is short, further study is needed, and the study is a retrospective single-center study, which may have selection bias.

## 1. Introduction

**Figure 1.**
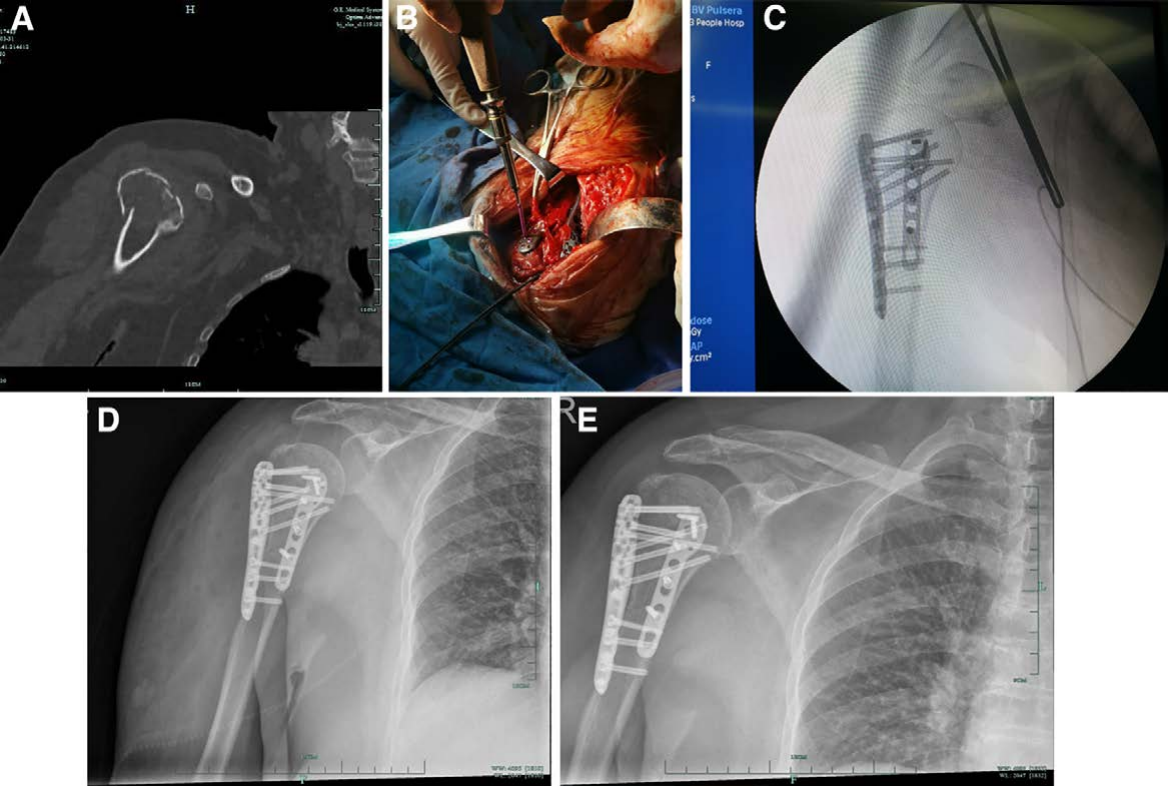
Typical case 1. (A) Preoperative computed tomography of the shoulder revealed a 70-year-old woman with comminuted and displaced fracture of the right proximal humerus. (B) Intraoperative image: after satisfactory reduction of the comminuted fracture of the proximal humerus, fixation with a proximal humerus locking plate was performed 0.5 to 1 cm below the greater tubercle of the humerus. Then, the anterior medial humerus was exposed after the upper limb abduction and external rotation, the antero-medial support plate was inserted medial to the intertubercular groove, and the screws were inserted. (C) Intraoperative X-ray image: Anteromedial support plate combined with lateral locking compression plate fixed the proximal humerus fracture, the fracture reduction was satisfactory, and the internal fixation position were good. (D) Postoperative shoulder joint X-ray: the reduction of the proximal humeral fracture was satisfactory, the position of the anteromedial support plate and lateral locking compression plate were good, and the length of the screws were good. (E) Six months after surgery shoulder joint X-ray: the proximal humerus fracture healed. There was no necrosis or varus deformity of the humerus. The position of the double plates were good, and there were no loosening or breakage of the screws.

Proximal humerus fractures (PHFs) are common in clinical practice, accounting for about 5% of all fractures.^[1]^ Unstable and displaced fractures, especially partial fractures of Neer III and IV, often is required surgical treatment, and the proximal lateral locking compression plate is usually used for internal fixation.^[2,3]^ In recent years, with the aggravation of aging, the incidence of PHFs in the elderly is also increasing year by year. The fractures are mostly comminuted, poor bone quality, and defects of the medial column of the proximal humerus (Fig. 1A). Therefore, the treatment of comminuted PHFs is still a challenge. It has been reported that there were many postoperative complications, such as loosening of internal fixation, internal rotation of the humerus head, necrosis of the humerus bone, failure of internal fixation, insertion of the humerus screw, acromion impact, etc.^[4]^ These complications were usually found in patients with loss of function of the proximal medial humerus column. Therefore, a number of clinical methods have been developed to reconstruct the function of the internal humerus column, such as fibula bone grafting, support plate, filling the humerus head with bone cement and so on. In the study, 31 eligible cases with comminuted PHFs between January 2017 and April 2023 were retrospectively analyzed. The objective is to evaluate the clinical efficacy of anteromedial support plate combined with lateral locking compression plate for the treatment of proximal humeral fractures. The report is as follows.

## 2. Data and methods

### 2.1. Patient inclusion and exclusion criteria

Patients were included in the study according to the following criteria: comminuted proximal humeral fractures (Neer partial III, Neer partial IV), acute closed fracture, shoulder joint function well before the injury, consent for surgery, and timely follow-up. Patients with any of the following conditions were excluded: old fractures, open fractures, pathological fractures, pain in the shoulder before injury, severe shoulder arthritis or joint deformity. Patients with the nervous system disease or mental illness, unable to follow up in time.

### 2.2. General information

A retrospective analysis was conducted on 31 eligible cases of comminuted PHFs from January 2017 to April 2023, including 12 males and 19 females. There were 11 cases on the left side and 20 on the right side. There were 21 cases of partial fracture of Neer III and 10 cases of partial fracture of IV. Causes of injury: 18 cases of falling injuries, 10 cases of traffic injuries, and 3 cases of other traumatic mechanism. Twelve cases with associated injuries: 7 cases in the single plate group (rotator cuff tear in 4 cases, dislocation of humeral head in 1 case, rib fracture in 1 case, facial fracture in 1 case); 5 cases in the double plate group (rotator cuff tear in 3 cases, humerus head dislocation in 2 cases). Fourteen cases were treated with anteromedial support plate combined with lateral locking compression plate of proximal humerus. Seventeen cases were treated with lateral locking compression plate of proximal humerus. This study was approved by Hangzhou Third People’s Hospital Medical Ethics Review Committee (2024KA021). Informed consent was obtained from all patients and/or their legal guardian(s) (Table 1 for details).

**Table 1:** Preoperative general data.

Group	Cases (n)	Gender (F/M)	Age (y)	Side (L/R)	Neer (III/IV)	Combined injuries (n)	Waiting time for operation (*d*)
Single plate	17	11/6	65.88 ± 10.40	5/12	12/5	7	3.94 ± 0.90
Double plate	14	8/6	67.64 ± 8.08	6/8	9/5	5	3.71 ± 1.27
		*X*^2^ = 0.004		*X*^2^ = 0.16	*X*^2^ = 0.00		*t* = 0.56
P*		.95	.60	.69	1.00		.58

*P < .05 is statistically significant.

## 3. Treatment methods

### 3.1. Surgical methods

After general anesthesia, the half-seated position was adopted. The operation area was routinely disinfected and covered with towels. The incision length of the anterolateral proximal humerus was about 10 to 12 cm. The skin and subcutaneous tissue were cut. The cephalic vein was exposed and protected. The fracture was fully exposed through blunt separation in the gap (between the deltoid and pectoralis major muscles). Firstly, the upper limb was placed in an abductive and internal rotation position to confirm the location of the greater tuberosity of the humerus and perform reduction. If the fracture was comminuted and the reduction was difficult, multiple strands of 5# Ethibond stitches were used to suture the tendon bone joint through the rotator cuff tissue, and the bone block was pulled downward for reduction. The rotator cuff tear was sutured and fixed.Fractures with significant bone defects can be treated by implanting allogeneic bone. 2 to 3 Kirschner wires were used for temporary fixation. After satisfactory reduction of the fracture was determined by X-ray, the proximal lateral humerus locking plate was placed 0.5 to 1 cm below the greater tubercle of the humerus, and the screws were successively inserted for fixation. The stitches were fixed to the positioning hole at the top of the plate. Then, the tubercle of the humerus was exposed after the upper limb abduction and external rotation. Fractures of the small tuberosity humerus and the medial column could be reduced and fixed in the same way. The anteromedial support plate was inserted medial to the intertubercular groove, and screws were inserted (Fig. 1B). Satisfactory fracture reduction and internal fixation were confirmed by X-ray again (Fig. 1C and D). Finally, the shoulder joint was moved to ensure that the fracture was stable and without impact. If the long-head tendon was injured or trapped by the plate, it should be cut and sutured to the upper margin of the pectoralis major. Abundant wound irrigation, placed the drainage tube, and layered suture.

### 3.2. Postoperative management

Prophylactic antibiotics were used 30 minutes before surgery and 1 day after surgery. The drainage tube was removed 48 hours after surgery. Shoulder pendulum movement the next day after surgery. After 1 to 2 weeks of surgery, active shoulder joint activity is feasible. At the same time to strengthen the wrist joint, elbow joint activity. From the third week after surgery, the range of motion of shoulder joint should be increased, such as shoulder lifting, internal rotation, external rotation, etc.

Routine outpatient follow-up was performed 1, 2, 3, 6, 9 months, 1 year, and 2 years after surgery. If X-ray show bone healing 3 months after surgery, some daily activities could be performed (Fig. 1E). Six months after surgery, the patient gradually returned to normal activities, physical labor, and contact sports. Patients were encouraged to increase their range of motion 6 weeks after surgery. If the X-ray shows bone healing 3 months after surgery, some daily activities can be carried out. Six months after surgery, patients gradually returned to normal activities, physical labor, and contact sports.

### 3.3. Scoring

Visual analogue scale (VAS) is a subjective assessment of pain degree by patients.^[5]^ The scale was 0 for no pain, 1 to 3 for mild pain, 4 to 6 for moderate pain, and 7 to 10 for severe pain. Used to assess the severity of shoulder pain.

The function of shoulder joint is evaluated by Constant–Murley score.^[5,6]^ The score includes pain (15 points), activities of daily living (20 points), active range of motion (40 points), and muscle strength (25 points) out of a total of 100 points. The higher the score, the better the function. The overall satisfaction is determined by Constant–Murley score: >75 is excellent, 50 to 75 is good, and <50 is unsatisfactory.

### 3.4. Statistical analysis

Statistical analysis is performed using SPSS software (version 22.0; IBM Corp., Armonk, NY, USA). Quantitative data are expressed as mean ± standard deviation (*x* ± s). Evaluation indicators: preoperative general data, intraoperative complications, operative time, intraoperative bleeding loss, postoperative complications, fracture healing time, changes in internal fixation, VAS 3 months postoperatively, Constant–Murley score 9 months postoperatively, etc.

## 4. Results

There was no statistical difference in preoperative general data between the 2 groups (*P* > .05), which was comparable (Table 1).

There were no major complications of vascular and nerve injury during the operation. Sixteen cases were filled with allogeneic bone (double plate group: 7 cases, single plate group: 9 cases).

All the incisions were healed in stage I. All patients were followed up for 6 to 24 months, with an average of 14.50 months. The fracture healing time in the double plate group was less than that in the single plate group, which was statistically significant (*P* < .05). There were no significant differences in operative time, bleeding loss, VAS score 3 months after surgery, and Constant–Murley score 9 months after surgery between the 2 groups (*P* > .05). Seven cases of postoperative complications: 2 cases in double plate group (1 case of humeral head collapse, 1 case of humeral head mild varus, no special treatment, no loosening of internal fixation) and 5 cases in single plate group (1 case of humeral head necrosis, 2 cases of humeral head varus, 1 case of acromial impingement, 1 case of screw withdrawal). The excellent and good rate of single plate group versus double plate group was 76.47% (13/17) versus 78.57% (11/14) (Table 2 for details).

**Table 2:** Perioperative and postoperative indicators.

Group (n)	Operation time (min)	Intraoperative bleeding loss (mL)	Bone graft (n)	Complications (n)	Fracture healing time (m)	VAS score	Constant–Murley score	Excellent and good rate
Single plate (17)	113.82 ± 10.23	158.24 ± 24.30	9	5	3.78 ± 0.38	3.06 ± 1.03	69.18 ± 15.14	76.47% (13/17)
Double plate (14)	120.36 ± 9.70	175.71 ± 27.93	7	2	3.46 ± 0.36	2.57 ± 0.76	76.43 ± 16.62	78.57% (11/14)
P*	.08	.08			.03	.14	.22	

VAS = visual analogue scale.

*P < .05 is statistically significant.

## 5. Discussion

### 5.1. Problems in the treatment of comminuted PHFs

With the aggravation of population aging, there will be more and more PHFs in the elderly, second only to hip and distal radius fractures.^[2–5]^ For most comminuted proximal humeral fractures, surgical reduction, and internal plate fixation are the main treatment methods (Fig. 1A). However, the traditional lateral proximal humeral plate has poor reduction and fixation effect on the humeral tubercles and medial humeral column fractures, and it cannot reconstruct the support function of the medial humeral column. Therefore, there is a high rate of surgical failure, such as loosening of internal fixation, screw withdrawal, humeral head varus, humeral head collapse, necrosis, nonunion of fractures, shoulder impingement, shoulder pain, etc. Complications were reported as high as 37.8%.^[6]^ Hardema et al^[7]^ reported that the incidence of reoperation after locking plate fixation in 122 patients with PHFs was 28%. Brunner et al^[8]^ found a complication rate of 35% after plate fixation in 158 patients with PHFs. In recent years, the concept of positive reduction of the proximal medial cortex of the humerus has been proposed, and it is considered to be one of the keys to the success of surgery.^[9–12]^ For the reason, some prescriptions have appeared, such as fibula bone grafting, bone cement filling of the humerus head, medial plate, etc. Due to the difficulty in operation, fibula transplantation is difficult to popularize. Cement-filled humerus head is not suitable for young and middle-aged patients and bone cement affects fracture healing. The both methods are difficult to use widely. Therefore, the treatment of comminuted PHFs has always been a worthy topic.

### 5.2. To evaluate the early clinical efficacy of anteromedial support plate for the treatment of comminuted fractures of proximal humerus

In recent years, more and more techniques of double plate fixation for PHFs have been used, with satisfactory results reported. In the group of 31 cases of comminuted proximal humeral fractures, 14 cases were treated with double plate fixation, and 17 cases with single plate fixation. Through a retrospective analysis of the treatment of comminuted proximal humeral fractures with anteromedial support plate, we find that there are certain advantages: first, using anteromedial support plate, the operation is simple and safe. The 2 plates are placed in the same incision and no other injuries are required during the operation. After fixation with a lateral plate, the upper limb was placed in external rotation to fully expose the small tuberosity and the medial side of the humeral neck. The suitable support plate can be inserted from the upper margin of latissimus dorsi and fixed to the medial margin of the intertubercular sulcus. Therefore, it does not significantly increase the operation time. According to the statistical results, the operation time of the double plate group is slightly longer than that of the single plate group, but statistically insignificant (*P* = .12). When placing the anterior medial support plate, the anterior medial soft tissue is bluntly separated and hemostasis is performed, which will not significantly increase intraoperative bleeding loss. This can be seen in the intraoperative bleeding loss statistics of the 2 groups (*P* > .05). Second, the fixation strength of fracture is increased with the addition of anteromedial support plate. In terms of fracture healing time, the double plate fixation group was superior to the single plate fixation group, and there was statistical significance (*P* = .03). The main reason is that the bilateral plates are fixed at the lateral and medial side of the proximal humerus at the same time, which effectively increased the fracture stability and reduced the fracture micromotion during shoulder joint activity (Fig. 1D). Fracture micromotion is the main cause of fracture nonunion, fracture displacement, and internal fixation loosening. After double plate fixation, it can reduce the postoperative fixation time and increase the range of joint motion, which is conducive to the postoperative functional recovery of shoulder joint. In the group, the shoulder joint recovery in the double plate group was better than that in the other group, although there was no statistically significant difference. Theoretically, with the increase of cases and extended follow-up time, the recovery of shoulder function in the double plate group should be statistically better than that in the single-plate group, which requires further study. Third, double plate fixation can reduce postoperative complications. Anteromedialsupport plate fixation not only increases the stability of fracture fixation, but also reduces postoperative complications. In the group, only 1 case of humeral head collapse and 1 case of humeral head mild varus occurred in the double plate group, which was significantly lower than the 5 cases of postoperative complications in the single plate group (1 case of humeral head necrosis, 2 cases of varus healing, 1 case of acromial impact, and 1 case with screw withdrawal). If the number of cases increases, the advantage of low postoperative complications in the double-plate group will become more and more obvious. Of course, the choice of anteromedial plate is also very important, such as T-shaped plate, ordinary compression plate, metacarpophalangeal miniature plate, etc. We preferred the distal fibula anatomical plate as the medial support plate. The anatomical plate of the distal fibula is thinner, which can reduce the occurrence of internal impingement. The distal end of the plate can be matched to the curvature of the humeral head. Multiple screws can be inserted at the distal end of the plate to increase stability.

### 5.3. Deficiencies and prospects

Dual plates fixation in the treatment of PHFs still presents some problems. The most obvious is that dual plates increase the cost. There is no uniform anteromedial plate of proximal humeral. In addition, the number of cases in this group was small and the follow-up time was short. Further accumulation of cases and further studies are needed. Moreover, the report is a single-center retrospective study, so there is a potential selection bias.

## Author contributions

**Data curation:** Wentao Lei.

**Methodology:** Guobiao Pan.

**Validation:** Lingzhi Ni.

**Writing – original draft:** Zhengfeng Mei.

**Writing – review & editing:** Zhengfeng Mei.
